# Biofilms grown in aquatic microcosms affect mercury and selenium accumulation in *Daphnia*

**DOI:** 10.1007/s10646-020-02194-4

**Published:** 2020-04-15

**Authors:** Semona Issa, Tomasz Maciej Ciesielski, Øyvind Mikkelsen, Sigurd Einum, Veerle L. B. Jaspers

**Affiliations:** 1 Department of Biology, Centre for Biodiversity Dynamics (CBD), Norwegian University of Science and Technology, Høgskoleringen 5, 7491 Trondheim, Norway; 2 Department of Biology, Norwegian University of Science and Technology, Høgskoleringen 5, 7491 Trondheim, Norway; 3 Department of Chemistry, Norwegian University of Science and Technology, Høgskoleringen 5, 7491 Trondheim, Norway

**Keywords:** Ecotoxicity testing, Trophic transfer, Metal exposure, Biotic interaction

## Abstract

Experiments examining mercury (Hg) toxicity in *Daphnia* are usually conducted in highly standardized conditions that prevent the formation of biofilm. Although such standardization has many advantages, extrapolation of results to natural conditions and inference of ecological effects is challenging. This is especially true since biofilms can accumulate metals/metalloids and play a key role in their transfer to higher trophic level organisms. In this study, we experimentally tested the effects of spontaneously appearing biofilm in *Daphnia* cultures on accumulation of Hg and its natural antagonist selenium (Se) in *Daphnia magna*. We added Hg (in the form of mercury (II) chloride) at two concentrations (0.2 µg/L and 2 µg/L) to experimental microcosms and measured the uptake of Hg and Se by *D. magna* in the presence and absence of biofilm. To test for consistent and replicable results, we ran two identical experimental sets one week apart. Biofilm presence significantly reduced the accumulation of Hg, while increasing the tissue Se content in *D. magna*, and these findings were reproducible across experimental sets. These findings indicate that highly standardized tests may not be adequate to predict the bioaccumulation and potential toxicity of metals/metalloids under natural conditions.

## Introduction

Lab-based aquatic toxicity tests are usually conducted under highly standardized conditions (OECD 1992, 2004), which allows for comparisons of toxicity of different compounds from experiments run in different laboratories and during different times. Yet, extrapolating results of such studies to natural populations is challenging, as they overlook the potential for interactions with other components in the biotic community to influence the effects of toxins on focal organisms (Holmstrup et al. 2010; Bone et al. 2012). *Daphnia*, freshwater zooplankton, are highly suitable for and widely used as model organisms for standardized tests to infer toxicity thresholds of aquatic organisms (Shaw et al. 2008). They are keystone grazers of phytoplankton as well as known consumers of bacteria and fungi (Kagami et al. 2004; Eckert and Pernthaler 2014). Understanding how toxic compounds affect the fitness of *Daphnia* is essential to understand the ecological consequences of pollution in freshwater ecosystems.

The inclusion of biotic interactions between *Daphnia* and biofilm in toxicity tests could provide a more ecologically realistic approach. Biofilms are aggregates of microorganisms (algae, cyanobacteria, bacteria, fungi, and protozoa) growing on surfaces and embedded in a matrix of extracellular polymeric substances (EPS; Decho 2000). In aquatic environments, biofilms are involved in organic matter cycling, primary production and respiration (Wetzel 1993; Kühl et al. 1996; Decho 2000). They serve as food to higher trophic levels through grazing (Huws et al. 2005; Siehoff et al. 2009), and their algal components exude organic carbon to be taken up by bacteria (Søndergaard et al. 1995; Goto et al. 2001). Despite the role played by biofilms in the aquatic food web, classic toxicity tests apply standardized methods that hinder their growth in exposure media (OECD 2012).

One aspect of biofilms that makes them potentially relevant for toxicity studies is the fact that they efficiently accumulate metals. This makes biofilms useful for monitoring metal contamination in aquatic ecosystems (Leguay et al. 2016), but may also influence the exposure experienced by higher trophic levels (Stewart et al. 2004; Cardoso et al. 2013). For example, aquatic biofilms can readily accumulate mercury (Hg) (Dranguet et al. 2017), as well as its natural antagonist selenium (Se) (Janz et al. 2014). In media and living organisms, selenide ions (Se^2−^) can bind to mercuric ions (Hg^2+^) to form mercuric selenide (HgSe), a stable and biologically inert complex, thereby reducing the risk of Hg toxic effects. Se/Hg molar ratios in organisms that approach or exceed one indicate that toxic effects of Hg are likely counteracted by Se (Yang et al. 2008). Successful accumulation of Hg and Se in biofilms may in turn affect their accumulation in *Daphnia*.

In this study, we experimentally tested for the effect of biofilm presence on Hg and Se uptake by *Daphnia magna*. Biofilm is an important food source for *Daphnia* (Siehoff et al. 2009) and can provide aquatic animals with an additional source of both Hg and Se through grazing. Because diet is the main source of Se accumulation in aquatic animals (Sandholm et al. 1973), biofilm is expected to have a positive effect on Se bioaccumulation. On the other hand, direct uptake of Hg by biofilm could reduce the amount of aqueous Hg available for uptake by animals (Ayangbenro and Babalola 2017), but may increase their dietary uptake of Hg. Hence, biofilms may play a central role in the transfer of both Hg and Se to higher trophic levels, possibly altering the effects of Hg pollution in aquatic ecosystems. Given the high international concern for Hg, due to its long-range transport across the globe and its various toxic properties (UNEP 2013), knowledge about how ecological interactions between *Daphnia* and biofilms may influence the relative uptake of Hg and Se and hence influence toxicity levels, seems crucial, but is currently lacking.

## Materials and methods

### Study organisms

Ephippia containing resting eggs resulting from sexual reproduction of *D. magna* were collected in November 2014, in a pond at Værøy Island (1·0 ha, 67·687°N 12·672°E), northern Norway. Ephippial eggs were hatched in the laboratory and propagated clonally. For this experiment, juveniles of a single clone (clone 47) of *D. magna* were asexually propagated for eight successive generations prior to use. *D. magna* were cultured in 2.5 L aquaria at 20 °C in a modified “Aachener Daphnien Medium” (ADaM) (Klüttgen et al. 1994, SeO_2_ concentration reduced by 50%), under long photoperiods (16 h L: 8 h D) using white fluorescent lamps. The medium was exchanged weekly and the animals were fed three times a week with Shellfish Diet 1800® (Reed mariculture Inc.; Rikard and Walton 2012) at a final concentration of 3.2 × 10^5^ algal cells/mL.

### Experimental design

**Fig. 1:**
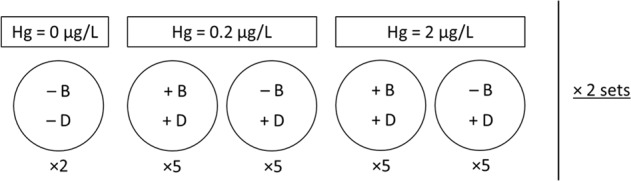
Schematic diagram of the experimental design. ×2 sets: two identical experimental sets were run one week apart. Two replicates per set for the control (×2), 5 replicates per treatment (×5). Treatments are defined by the Hg concentration (0 μg/L Hg versus 0.2 μg/L Hg and 2 μg/L Hg), presence of biofilm (absent (−B) versus present (+B)) and presence of *Daphnia* (absent (−D) versus present (+D))

To test for consistent and replicable results, we ran two identical experimental sets (Fig. 1), one week apart, where we allowed for the spontaneous growth of biofilm in the *Daphnia* culture medium, which may have resulted in differences in biofilm composition between the experimental sets.

For each experimental set, a full factorial design with two different starting concentrations of Hg (0.2 µg/L and 2 µg/L) and two biofilm treatments (present or absent) was applied, with five replicate beakers for each of the four combinations. In addition, two blanks containing only ADaM medium were used parallel to each experimental set and were treated to the same conditions as the exposure beakers. The two exposure concentrations (0.2 µg/L and 2 µg/L) were selected for being non-lethal and environmentally relevant concentrations of Hg based on literature research (Table A.1). Hg stock solutions (0.0016 g/L) were prepared at the onset of each experimental set, by dissolving 99.5% pure mercury (II) chloride (HgCl_2_) (Fluka, Switzerland) in Milli-Q water (18.2 MΩ cm) (Milli-Q Plus, Millipore Corp.). The exposure glass beakers and equipment used for making Hg stock solutions were acid-washed overnight before use with 1 M HNO_3_ suprapure quality prepared with a sub-boiling distillation system (Milestone, SubPUR) and subsequently washed with Milli-Q water. The stock solutions were added to ADaM to create the desired Hg exposure concentrations. For the biofilm absence treatment, glass beakers were used immediately after the cleaning procedure was completed. For the biofilm presence treatment, glass beakers were allowed to develop biofilm on their walls, in the presence of ten juveniles of a single clone of *D. magna* (clone 47), during two weeks prior to the experiment. Moreover, a total of eight controls containing only ADaM were added parallel to the beakers with biofilm and were subjected to identical biofilm growth conditions.

In each experimental replicate beaker (600 mL non-aerated borosilicate beakers, Fisherbrand), 30 adults of the same clone were kept in 400 mL medium at 20 °C for a period of 96 h. The ADaM medium was not replaced during the experimental period, and the animals were maintained under long photoperiods (16 h L: 8 h D) and fed with Shellfish Diet 1800® on days 1 and 3 of each experimental set at a final concentration of 3.2 × 10^5^ algal cells/mL.

### Sampling procedure

On the third day of the biofilm growth period, we sampled the medium in the biofilm-containing beakers and their associated blanks (*n* = 28) for Hg and Se analysis. On the first and last experimental days, we sampled 100 mL of medium for measurements of pH (WTW pH 340i), conductivity (WTW LF 330 conductivity meter), and dissolved oxygen (WTW Multi 3410 multiprobe meter), and took 5 mL of medium for measurements of Hg, Se, chloride (Cl), calcium (Ca) and magnesium (Mg) (*n* = 88; experimental beakers and their associated blanks). In addition, ADaM used for Hg dilution and exposure was sampled (*n* = 4) on the first experimental days for testing of Hg and Se levels. Immediately after collection, all medium samples (*N* = 120) were filtered through 25 mm diameter polyethersulphone membrane (0.45 μm) disposable syringe filters (VWR International) and acidified to 0.1 M HNO_3_ (Milestone, SubPUR). Prior to sample withdrawal, the syringe filters were flushed with a few mL of clean ADaM and subsequently with the medium. Ca and Mg concentrations were used to calculate Ca hardness as follows: CaCO_3_ (mg/L) = 2.5 Ca (mg/L) + 4.1 Mg (mg/L).

To obtain tissue blanks for Hg and Se analysis, animals were collected from the *Daphnia* cultures (clone 47) prior to the start of each experimental set. For analysis of final tissue Hg and Se content, animals were sampled on the last experimental day, and then washed in a new culture medium for 5 min to remove Hg from the carapace fluid. Finally, all tissue samples (*N* = 44) were freeze-dried and stored at room temperature until further analysis (for more details on quality control check Table A.2).

### Chemical analysis

The medium samples were analyzed using high resolution inductively coupled plasma mass spectrometry (HR-ICP-MS, Element 2, Thermo-Fisher Scientific). The freeze-dried tissue samples (6.30 ± 0.12 mg dry mass per replicate beaker, mean ± SE) were first acid digested using 3 mL 50% HNO_3_ per tissue sample (Milestone, SubPUR) in a high-pressure microwave system (Milestone UltraClave, EMLS, Leutkirch Germany) according to a temperature profile that increases gradually from room temperature up to 250 °C within 1 h, followed by a cooling step that allows temperature to return back to its initial value within ca. 1 h. After cooling to room temperature, the digested samples were diluted with Milli-Q water (18.2 MΩ cm) (Milli-Q Plus, Millipore Corp.) in polypropylene vials to achieve a final HNO_3_ concentration of 0.6 M. Finally, samples were analyzed with HR-ICP-MS and the tissue Hg and Se content was calculated on a dry-mass basis. Seven blank samples containing Milli-Q water and HNO_3_ (0.6 M in final solution) were run parallel to the digestion of the tissue samples. Results were corrected for reagent blank values. Certified reference material Polish Virginia Tobacco Leaves (INCT-PVTL-6) (Samczyński et al. 2012) was used to verify the accuracy of the Hg and Se analysis. The mean concentrations found (0.0264 ± 0.0006 μg/g dry weight) were in good agreement with the certified values (0.0232 ± 0.0016 μg/g dry weight).

### Statistical analysis

All statistical analyses and graphic illustrations were performed in R v. 3.4.3. (R Development Core Team 2016). For exposure and water quality variables (Hg, Se, Cl, calcium hardness, dissolved oxygen, conductivity and pH), summary statistics (mean ± standard error) were calculated and full models were fitted using Hg concentration, biofilm and experimental set (hereafter “set”) as fixed predictor variables and replicate beaker as a random predictor variable. For tissue Hg and Se content and Se/Hg molar ratios in *Daphnia*, full models with fixed predictor variables being Hg concentration, biofilm and set were fitted.

The models were implemented using the *lme* and *gls* functions in the package *nlme* (Pinheiro et al. 2018). Model selection followed a backwards selection procedure, where variables were removed sequentially, starting with random effects, using likelihood ratio tests (Zuur et al. 2009). Model residuals were checked for homogeneous variance and for normal distribution. The VarIdent command from the *nlme* package was used to allow residual variance to differ among Hg concentrations, biofilm levels and sets. Tukey’s multiple comparison test was implemented where groups were significantly different. We used a significance level *α* = 0.05 for hypothesis testing.

## Results

No mortality occurred during the exposure. The Hg concentration was significantly higher in media without biofilm compared to media with biofilm that were exposed to 2 μg/L Hg in set 1 only (Table 1). Se in the medium, pH and conductivity significantly decreased in the presence of biofilm (Table 1). Furthermore, Cl, hardness and conductivity were significantly higher in set 1 compared to set 2 (Table 1). The opposite was true for pH and dissolved oxygen (Table 1). In addition, dissolved oxygen significantly increased in the presence of biofilm in set 1 only (Table 1). Nevertheless, despite significant effects of treatments and/or sets on conductivity, dissolved oxygen, pH, Cl and hardness, the magnitude of change in these measures was relatively small. Indeed, pH, conductivity and Cl varied by a maximum of 5%, hardness by 8% and dissolved oxygen by 11% (Table 1).

**Table 1:** Exposure and water quality variable averages (averaged over the first and last experimental days) are compared across all sets and treatment combinations

	Treatment							
	Biofilm present 0.2 Hg (µg/L)		Biofilm absent 0.2 Hg (µg/L)		Biofilm present 2 Hg (µg/L)		Biofilm absent 2 Hg (µg/L)	
Set	1	2	1	2	1	2	1	2
Hg^2+^ (µg/L)	0.024 ± 0.003^a^	0.026 ± 0.003^a^	0.050 ± 0.01^a^	0.027 ± 0.01^a^	0.12 ± 0.01^a^	0.15 ± 0.02^a^	0.51 ± 0.1^b^	0.14 ± 0.03^a^
Se^2−^ (µg/L)	5.8 ± 0.05^b^	5.7 ± 0.1^b^	5.9 ± 0.05^a^	5.9 ± 0.09^a^	5.7 ± 0.06^b^	5.7 ± 0.07^b^	5.9 ± 0.04^a^	5.7 ± 0.04^a^
Conductivity (mS/cm)	2.2 ± 0.004^bc^	2.1 ± 0.01^bd^	2.2 ± 0.01^ac^	2.2 ± 0.01^ad^	2.2 ± 0.004^bc^	2.1 ± 0.01^bd^	2.3 ± 0.002^ac^	2.1 ± 0.01^ad^
Dissolved oxygen (mg/L)	7.9 ± 0.05^bc^	8.1 ± 0.1^bd^	7.5 ± 0.08^a^	8.2 ± 0.06^bd^	7.8 ± 0.1^bc^	8.2 ± 0.1^bd^	7.6 ± 0.02^a^	8.3 ± 0.1^bd^
pH	7.7 ± 0.02^bc^	7.8 ± 0.04^bd^	7.8 ± 0.04^ac^	7.9 ± 0.02^ad^	7.7 ± 0.02^bc^	7.9 ± 0.05^bd^	7.8 ± 0.05^ac^	8.0 ± 0.08^ad^
Ca hardness (mg/L)	350 ± 3^a^	320 ± 10^b^	340 ± 5^a^	340 ± 4^b^	340 ± 5^a^	320 ± 8^b^	340 ± 3^a^	330 ± 8^b^
Cl^−^ (mg/L)	630 ± 4^a^	605 ± 9^b^	630 ± 6^a^	610 ± 8^b^	630 ± 3^a^	602 ± 6^b^	630 ± 3^a^	610 ± 6^b^

Values are given as mean ± SE. Means with the same letter are not significantly different from each other

**Fig. 2:**
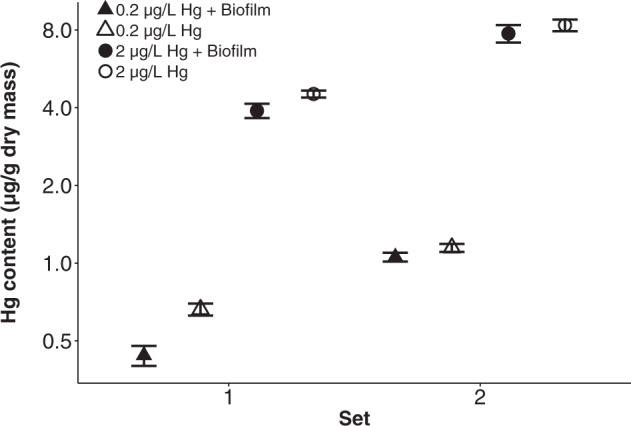
Tissue Hg content (µg/g dry mass) in *Daphnia* in response to growth medium Hg concentrations, biofilm presence versus absence and set (mean ± SE). The y-axis is on a logarithmic scale with base 2

**Fig. 3:**
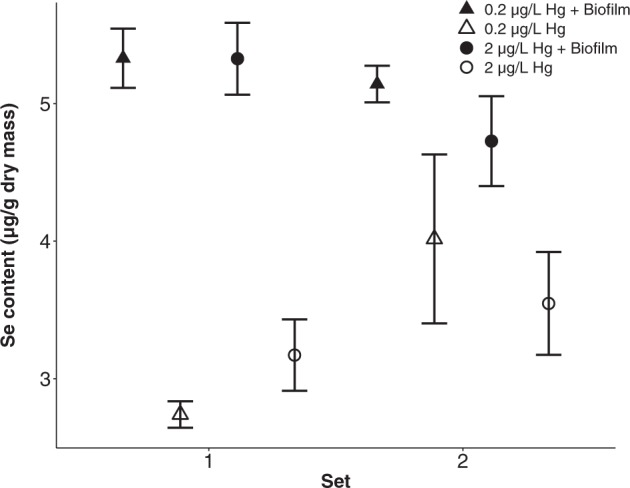
Tissue Se content (µg/g dry mass) in *Daphnia* in response to growth medium Hg concentrations, biofilm presence versus absence and set (mean ± SE). The y-axis is on a linear scale

**Fig. 4:**
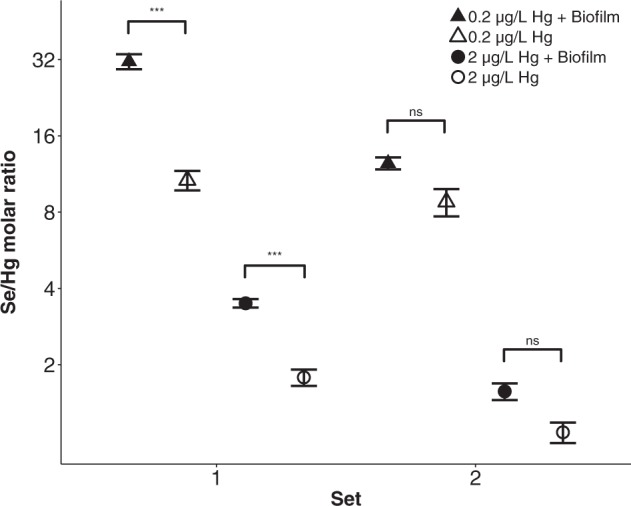
Se/Hg molar ratios in *Daphnia* in response to growth medium Hg concentrations, biofilm presence versus absence and set (mean ± SE). Significance was evaluated using the Tukey’s HSD post hoc test. Significant differences were observed between the two biofilm treatments for set 1 only (***P < 0.001; ns not significant). The y-axis is on a logarithmic scale with base 2

Tissue Hg content was significantly higher in daphnids exposed to 2 μg/L Hg compared to 0.2 μg/L Hg (6.1 ± 0.5 μg/g dry mass versus 0.8 ± 0.07 μg/g dry mass, *P* < 0.001) and approximately twofold higher in set 2 compared to set 1 (4.6 ± 0.8 μg/g dry mass versus 2.4 ± 0.4 μg/g dry mass, *P* < 0.001) (Fig. 2). In addition, biofilm presence significantly reduced the overall *Daphnia* tissue Hg content from 0.9 ± 0.1 to 0.7 ± 0.1 μg/g dry mass at 0.2 μg/L Hg, and from 6.4 ± 0.7 to 5.8 ± 0.7 μg/g dry mass at 2 μg/L Hg (*P* < 0.001) (Fig. 2). On the other hand, the overall *Daphnia* tissue Se content significantly increased in the presence of biofilm from 3.4 ± 0.2 μg/g dry mass to 5.1 ± 0.1 μg/g dry mass (*P* < 0.001) (Fig. 3). Consequently, Se/Hg molar ratios significantly increased in the presence of biofilm in set 1 from 11 ± 0.9 (no biofilm) to 31 ± 2.1 (with biofilm; *P* < 0.001) under exposure to 0.2 μg/L Hg and from 1.8 ± 0.1 (no biofilm) to 3.5 ± 0.1 (with biofilm; *P* < 0.001) under exposure to 2 μg/L Hg. However, biofilm presence did not significantly affect molar ratios in set 2 (12 ± 0.7 versus 8.8 ± 1.1 at 0.2 μg/L Hg, ns, and 1.6 ± 0.1 versus 1.1 ± 0.1 at 2 μg/L Hg, ns) (Fig. 4). More detailed results can be found in the Supplementary Material Tables A.3 and A.4.

## Discussion

In this study, we examined whether biofilm could affect Hg and Se accumulation in *Daphnia magna* through aqueous and dietary uptake pathways. The Hg exposure concentrations used were lower than the acute LC_50_ of 2.2 µg/L Hg in cladocerans (Nichols et al. 1997), although exceeding mean total concentrations of 0.006 µg/L typically found in the aquatic environment (Chen et al. 2000). Conductivity, dissolved oxygen and pH were also within the recommended range for testing metals in OECD test protocols for this species (OECD 2004). While hardness exceeded the recommended range, hardness has a negligible effect on Hg toxicity, in contrast to other heavy metals (Rathore and Khangarot 2003). Overall, there was no observed *Daphnia* mortality from either Hg exposure or changes in water quality.

There was a clear difference in Hg bioaccumulation between experimental sets, with higher tissue Hg content in set 2 compared to set 1. This was not due to differences in medium Hg concentrations but was possibly related to the higher medium Cl concentrations in set 1. Increased Cl has been shown to reduce the bioavailability of inorganic Hg through speciation (Wang and Wang 2010). In addition, dissolved organic carbon (DOC) could differ between sets, although this was not assessed in the current study. It is therefore important to always record and consider the potential influence of water quality variables when interpreting results from experimental exposure studies in aquatic microcosms. Moreover, differences between sets could be due to a potential difference in *Daphnia* age, albeit unlikely, and evidence to support this is currently lacking.

*Daphnia* tissue analysis showed that the increase in Hg content with increasing medium Hg concentrations was less significant in the presence of biofilm. Furthermore, biofilm presence increased *Daphnia* tissue Se content. These findings were replicable across experimental sets, which is important to highlight, as consistent and replicable effects are critical for improving risk analysis approaches (National Research Council 2009).

Previous research has provided evidence of aquatic biofilms accumulating heavy metals (Hill and Larsen 2005; Kohušová et al. 2011), through rapid absorption and removal from the aqueous solution (Chang et al. 2006; Ancion et al. 2010). This is because biofilms have a high capacity to absorb metals, which makes them a useful bioremediation tool (Dixit et al. 2015). Therefore, the biofilm in our study may potentially have acted as an available dietary source of Hg to the *Daphnia*, which could however not be confirmed as we were unable to analyze the Hg and Se content in the whole biofilm in this study. To determine the total mass balance of Hg and Se (total mass of biofilm and its Hg and Se content) in the exposure beakers, we would need to sample the entirety of the biofilm from the beakers. This was unfortunately not possible as it adhered to the walls and could not be entirely scraped off. Future studies should consider the possibility of growing the biofilm in filters placed inside the beakers at the start of the experiment for collection later. However, Tsui and Wang (2004) showed that *D. magna* accumulated Hg(II) mainly from the aqueous phase, through absorption, rather than from the ingestion of Hg enriched food. In the current study, uptake of aqueous Hg by daphnids may have been more important than dietary uptake as the presence of biofilm decreased the accumulation of Hg in the *Daphnia*. Indeed, biofilm may have indirectly reduced Hg accumulation in the animals, by reducing the amount of bioavailable aqueous Hg. However, this is not supported by our measurements in the medium, as observed changes in medium Hg concentrations (Table 1) do not suggest extensive uptake of the metal by biofilm.

Alternatively, biofilms can further reduce metal accumulation at higher trophic levels through “bloom dilution”, whereby increased phytoplankton biomass reduces the concentration of metal per cell available to grazers (Pickhardt et al. 2002). Another phenomenon is “growth biodilution”, observed in rapidly growing phytoplankton, where the concentration of metals within cells is diluted by growth during the day (measured as photosynthetically fixed carbon) (Hill and Larsen 2005; Poste et al. 2015). Hill and Larsen (2005) showed how this phenomenon substantially decreased metal concentrations in biofilm within a short period of 4 days, equal to the duration of the current experiment. Thus, if biofilm was an important dietary source of Hg to the *Daphnia* in the current study, the animals would have been exposed to lower Hg concentrations from diet than from the aqueous phase because of “growth biodilution”. However, it was not possible to determine unambiguously the main source of Hg to the daphnids in our study, as we were unable to determine the total mass balance of Hg (total mass of biofilm and its Hg content) in the exposure beakers.

Although the exact mode of action of biofilm presence on Hg accumulation in *Daphnia* is unknown, the presence of biofilm significantly reduced tissue Hg content and increased tissue Se content in *Daphnia*. The latter suggests dietary uptake of Se from biofilm. Lower Se concentrations measured in media with biofilm imply the metalloid’s uptake by biofilm microorganisms. Indeed, biofilm components are known to readily absorb Se under both laboratory and field conditions, rendering it bioavailable to higher trophic levels through diet (Fan et al. 2002; Ranjard et al. 2003; Tuzen and Sarı 2010). In agreement with the majority of studies that have investigated Se transfer in aquatic ecosystems (Stewart et al. 2004; Conley et al. 2009), our findings support that diet may be the predominant route of Se exposure for organisms in aquatic food webs. Nevertheless, at high aqueous Se concentrations, the proportion of dietary to direct Se uptake may decrease. This was previously detected in daphnids exposed to 31.6 µg/L Se (Guan and Wang 2004), a concentration that is however far above those observed in the current experiment.

Biofilm-induced changes in tissue Se and Hg content led to an increase in Se/Hg molar ratios in the animals, which remained above one in all treatments. This suggests that tissue Se content was probably high enough to counteract toxic effects of Hg in the *Daphnia* in the current experiment (Peterson et al. 2009).

In summary, the presence of biofilm reduced Hg accumulation in *Daphnia*. This reduction was probably not due to a reduction in dissolved Hg available to animals or to “growth biodilution” in the biofilm, as these processes would require significant uptake of Hg by the biofilm, which was not supported by observed changes in medium Hg concentrations. Therefore, the exact driver of the lower tissue Hg content in the presence of biofilm remains unknown and future analysis of biofilm content and DOC is strongly recommended. On the other hand, the presence of biofilm increased the accumulation of Se in the animals. Thus, biofilms could play a central role in the transfer of Se through the freshwater food web, subsequently providing potential protection against Hg toxicity in the animals. Thus, aquatic biofilms can affect the transfer of Hg and Se to grazing zooplankton, which then act as conduits of Hg and Se to subsequent higher trophic levels of the food web. Our findings support the observation that including natural variability in toxicity studies and allowing for food web interactions may be important for more realistic environmental exposure assessments (De Laender et al. 2008; Viaene et al. 2015). Therefore, in order to obtain ecologically relevant results, we recommend that aquatic toxicity studies on metals/metalloids should include interactions with biofilm components. However, further research is necessary to conclude on the main mechanism of Hg and Se accumulation in *Daphnia* in the presence of biofilm.

## Supplementary information

Supplementary Appendix 1

Supplementary Appendix 2
