# Supplementary material for: Biofilms grown in aquatic microcosms affect mercury and selenium accumulation in Daphnia
Source: Ecotoxicology. 2020 Apr 15;29(4):485–92. doi: 10.1007/s10646-020-02194-4 (PMC7182615; doi:10.1007/s10646-020-02194-4)
Supplement: Supplementary file 1 — Supplementary Appendix 1 [file 10646_2020_2194_MOESM1_ESM.doc]

**Biofilms grown in aquatic microcosms affect mercury and selenium accumulation in *Daphnia***

Semona Issa^1*^, Tomasz Maciej Ciesielski^2^, Øyvind Mikkelsen^3^, Sigurd Einum^1^, Veerle L. B. Jaspers^2^

^1^Centre for Biodiversity Dynamics (CBD), Department of Biology, Norwegian University of Science and Technology, Høgskoleringen 5, 7491 Trondheim, Norway

^2^Department of Biology, Norwegian University of Science and Technology, Høgskoleringen 5, 7491 Trondheim, Norway

^3^Department of Chemistry, Norwegian University of Science and Technology, Høgskoleringen 5, 7491 Trondheim, Norway

*E-mail contact: [semona.issa@ntnu.no](mailto:semona.issa@ntnu.no)

**Journal name:** *Ecotoxicology*

**Table A.1.** Studies examining Hg toxicity to *Daphnia* species.

| **Study** | **Test type** | **Exposure duration** | **Hg^2+^ exposure concentration** | **Life stage** | **Sample size** | **Endpoints** |
| --- | --- | --- | --- | --- | --- | --- |
| Tsui, M. T. K., & Wang, W.-X. (2005). Multigenerational acclimation of *Daphnia magna* to mercury: Relationships between biokinetics and toxicity. *Environ. Toxicol. Chem., 24*(11), 2927-2933. doi:10.1897/05-085R.1 | Static nonrenewal acute | 48 h | Control; 20; 40; 60; 80 and 105 µg/L | Juveniles (4 day old) and adults (21 day old) | 5 individuals/replicate  4 replicates/concentration level | LC_50_  Mortality |
|  | Static renewal chronic | 21 days | Control and 3.8 ± 0.7 μg/L | Juveniles (4 day old) | 100 individuals/replicate  3 replicates/concentration level | Biokinetics |
| Khangarot, B. S., & Das, S. (2009). Toxicity of mercury on in vitro development of parthenogenetic eggs of a freshwater cladoceran *Daphnia carinata*. *J. Hazard. Mater., 161*(1), 68-73. doi:10.1016/j.jhazmat.2008.03.068 | Static nonrenewal chronic and acute | 24-72 h | Control; 0.1; 0.32; 1; 3.2; 10 and 32 µg/L | Daphnid eggs (2-6 h old) | 5 replicates/concentration level | Mortality  Development time  EC_50_  Abnormalities in development |
| Biesinger, K. E., Anderson, L. E., & Eaton, J. G. (1982). Chronic effects of inorganic and organic mercury on *Daphnia magna*: Toxicity, accumulation, and loss. *Arch. Environ. Contam. Toxicol., 11*(6), 769-774. doi:10.1007/BF01059166 | Static renewal chronic | 21 days | Control; 0.85; 1.7; 3.4; 6.8 and 13.6 µg/L | Neonates (12 ± 12 h old) | 5 individuals/replicate  4 replicates/concentration level | Survival  Reproduction |
| Tsui, M. T. K., & Wang, W.-X. (2006). Acute toxicity of mercury to *Daphnia magna* under different conditions. *Environ. Sci. Technol., 40*(12), 4025-4030. doi:10.1021/es052377g | Static nonrenewal acute | 24 h | Control plus 5 to 8 concentrations ranging from 5 to 105 µg/L | Juveniles (4 day old) | 10-15 individuals/replicate  3 replicates/concentration level | LC_50_  Survival |
| Rodrigues, A. C. M., Jesus, F. T., Fernandes, M. A. F., Morgado, F., Soares, A. M. V. M., & Abreu, S. N. (2013). Mercury toxicity to freshwater organisms: Extrapolation using species sensitivity distribution. *Bull. Environ. Contam. Toxicol., 91*(2), 191-196. doi:10.1007/s00128-013-1029-0 | Static nonrenewal acute | 24-48 h | *D. magna*: control; 0.5; 1; 2; 4; 6; 8 and 16 µg/L  *D. longispina*: control; 0.5; 1; 3; 6; 9; 18 and 36 µg/L | Neonates (<24 h old) | 5 individuals/replicate  5 replicates/concentration level | 24 h EC_50_  48 h EC_50_  EC50 measured as immobilization |
| De Coen, W. M., Janssen, C. R., & Segner, H. (2001). The use of biomarkers in *Daphnia magna* toxicity testing V. *In vivo* alterations in the carbohydrate metabolism of *Daphnia magna* exposed to sublethal concentrations of mercury and lindane. *Ecotoxicol. Environ. Saf., 48*(3), 223-234. doi:10.1006/eesa.2000.2009 | Static renewal | 96 h | Control; 1.8; 3.2; 5.6; 10; 13 and 18 µg/L | Juveniles (<24 h) | 100 individuals/replicate  3 replicates/concentration level | Survival at 48 and 96 h  Enzymatic activity |
| De Coen, W. M., & Janssen, C. R. (1997). The use of biomarkers in *Daphnia magna* toxicity testing II. Digestive enzyme activity in *Daphnia magna* exposed to sublethal concentrations of cadmium, chromium and mercury. *Chemosphere, 35*(5), 1053-1067. doi:10.1016/S0045-6535(97)00172-0 | Static renewal chronic | 48-96 h | Control; 1.8; 3.2; 5.6; 10 and 30 µg/L | Juveniles (<24 h) | 3 replicates/concentration level | Enzymatic activity |
| De Coen, W. M., & Janssen, C. R. (1997). The use of biomarkers in *Daphnia magna* toxicity testing. IV. Cellular energy allocation: A new methodology to assess the energy budget of toxicant-stressed *Daphnia* populations. *J. Aquat. Ecosyst. Stress Recovery, 6*(1), 43-55. doi:10.1023/A:1008228517955 | Static renewal acute | 96 h | Control; 1.8; 3.2; 5.6; 10; 18; 24 and 32 µg/L | Juveniles (<24 h) | 11 individuals/replicate  3 replicates/concentration level | Energy budget  Growth (carapace length) |
|  | Static renewal chronic | 21 days | Control; 1.8; 3.2; 5.6; 10; 18; 24 and 32 µg/L | Juveniles (<24 h) | 10 individuals/replicate  4 replicates/concentration level | *r*_m_  Offspring number  Age-specific survival and reproduction |
| Meng, Q., Li, X., Feng, Q., & Cao, Z. (2008). *The acute and chronic toxicity of five heavy metals on the Daphnia magna*. Paper presented at the 2008 2nd International Conference on Bioinformatics and Biomedical Engineering, Shanghai, China. | Static renewal chronic | 14 days | Control and 0.5 µg/L | Neonates (6-12 h old) | 10 individuals/replicate  3 replicates/concentration level | Adult survival rate  Time to first reproduction  Number of juveniles per brood |

**Table A.2.** Summary of method blanks. Conductivity, dissolved oxygen, pH, hardness and Hg, Se and Cl ion concentrations are compared across all sets (1 versus 2). In the case of experimental blanks, these variables are averaged over the first and last experimental days for all replicates per set. Values are given as mean ± SE. n is the number of replicates.

|  | **Method blank type** | | | | | | | | | | |  |
| --- | --- | --- | --- | --- | --- | --- | --- | --- | --- | --- | --- | --- |
|  | Biofilm growth | | | | ADaM used for dilution and exposure (µg/L) | | Experimental blanks (µg/L) | | Tissue blanks  (µg/g) | | Shellfish Diet 1800® (µg/g) | |
|  | Beakers with animals (µg/L) | | Beakers without animals (µg/L) | |  | |  | |  | |  | |
| **Set** | 1 | 2 | 1 | 2 | 1 | 2 | 1 | 2 | 1 | 2 | - | |
| **Hg^2+^** | 0.00 ± 0.00  (n = 10) | 0.00 ± 0.00  (n = 10) | 0.01 ± 0.00  (n = 4) | 0.00 ± 0.00  (n = 4) | 0.00 ± 0.00  (n = 2) | 0.02 ± 0.00  (n = 2) | 0.01 ± 0.00  (n = 4) | 0.00 ± 0.00  (n = 4) | 0.00 ± 0.00  (n = 2) | 0.00 ± 0.00  (n = 2) | 0.00 ± 0.00  (n = 1) | |
| **Se^2-^** | 5.77 ± 0.06  (n = 10) | 5.77 ± 0.05  (n = 10) | 5.76 ± 0.01  (n = 4) | 5.72 ± 0.12  (n = 4) | 5.47 ± 0.15  (n = 2) | 5.65 ± 0.04  (n = 2) | 5.95 ± 0.11  (n = 4) | 6.09 ± 0.10  (n = 4) | 3.90 ± 0.24  (n = 2) | 3.95 ± 1.18  (n = 2) | - | |
| **Conductivity**  **(mS/cm)** | - | - | - | - | - | - | 2.25 ± 0.00  (n = 2) | 2.14 ± 0.01  (n = 4) | - | - | - | |
| **Dissolved oxygen**  **(mg/L)** | - | - | - | - | - | - | 8.75 ± 0.00  (n = 2) | 8.92 ± 0.04  (n = 4) | - | - | - | |
| **pH** | - | - | - | - | - | - | 7.98 ± 0.04  (n = 4) | 8.12 ± 0.10  (n = 4) | - | - | - | |
| **Ca hardness**  **(mg/L)** | - | - | - | - | - | - | 347 ± 5.88  (n = 4) | 346 ± 8.16  (n = 4) | - | - | - | |
| **Cl^–^**  **(mg/L)** | - | - | - | - | - | - | 648 ± 7.34  (n = 4) | 617 ± 12.0  (n = 4) | - | - | - | |

**Table A.3.** Model selection using AICc of candidate models for testing effects of Hg concentration (0.2 μg/L Hg(II) versus 2 μg/L Hg(II)), Biofilm (absent versus present) and Set (1 versus 2) on Hg and Se concentrations in the medium and their content in the animals; Se/Hg molar ratios in the animals; and Cl, calcium hardness, pH, conductivity and dissolved oxygen in the medium. Models were sorted by ΔAICc. The best random effect structure was first determined with REML on models that included all listed fixed effects. Fixed effects were then compared with ML using the best random effect structure. K is the number of parameters estimated. The least complex model within 2 ΔAICc is bolded.

| **Response variable** | **Model** | **K** | **AICc** | **∆AICc** | **wAICc** |
| --- | --- | --- | --- | --- | --- |
|  |  |  |  |  |  |
| **Hg in medium (µg/L)** |  |  |  |  |  |
| Fixed effects | **Hg _medium_ ~ Biofilm:Set:Hg** | 10 | -200.20 | 0.00 | 0.94 |
|  | Hg _medium_ ~ Biofilm:Set + Biofilm:Hg + Hg:Set | 9 | -193.20 | 6.98 | 0.03 |
|  | Hg _medium_ ~ Biofilm:Hg + Hg:Set | 8 | -190.50 | 9.65 | 0.01 |
|  | Hg _medium_ ~ Biofilm:Set + Biofilm:Hg | 8 | -190.40 | 9.72 | 0.01 |
|  | Hg _medium_ ~ Biofilm:Set + Hg:Set | 8 | -189.20 | 11.01 | 0.00 |
| Random effects | **vI (Hg)** | 10 | -151.80 | 0.00 | 0.79 |
|  | vI (Hg) + (1 \| Beaker) | 11 | -149.10 | 2.69 | 0.21 |
|  | vI (Biofilm) | 10 | -109.70 | 42.14 | 0.00 |
|  | vI (Biofilm) + (1 \| Beaker) | 11 | -107.00 | 44.83 | 0.00 |
|  | vI (Set) | 10 | -74.00 | 77.78 | 0.00 |
|  |  |  |  |  |  |
| **Se in medium (µg/L)** |  | **K** | **AICc** | **∆AICc** | **wAICc** |
| Fixed effects | **Se _medium_ ~ Biofilm** | 4 | -12.10 | 0.00 | 0.33 |
|  | Se _medium_ ~ Biofilm + Set | 5 | -11.00 | 1.12 | 0.19 |
|  | Se _medium_ ~ Biofilm + Hg | 5 | -10.40 | 1.76 | 0.14 |
|  | Se _medium_ ~ Biofilm + Set + Hg | 6 | -9.20 | 2.93 | 0.08 |
|  | Se ~ Biofilm:Set | 6 | -8.80 | 3.30 | 0.06 |
| Random effects | **vI (Set)** | 10 | 25.20 | 0.00 | 0.69 |
|  | vI (Set) + (1 \| Beaker) | 11 | 27.90 | 2.69 | 0.18 |
|  | vI (Hg) | 10 | 29.70 | 4.52 | 0.07 |
|  | vI (Biofilm) | 10 | 32.00 | 6.87 | 0.02 |
|  | vI (Hg) + ( 1\| Beaker) | 11 | 32.40 | 7.22 | 0.02 |
|  |  |  |  |  |  |
| **Cl in medium (mg/L)** |  | **K** | **AICc** | **∆AICc** | **wAICc** |
| Fixed effects | **Cl ~ Set** | 4 | 1794.20 | 0.00 | 0.44 |
|  | Cl ~ Biofilm + Set | 5 | 1796.30 | 2.04 | 0.16 |
|  | Cl ~ Set + Hg | 5 | 1796.40 | 2.20 | 0.14 |
|  | Cl ~ Biofilm:Set | 6 | 1797.60 | 3.43 | 0.08 |
|  | Cl ~ Biofilm + Set + Hg | 6 | 1798.50 | 4.30 | 0.05 |
| Random effects | **vI (Set)** | 10 | 1654.90 | 0.00 | 0.72 |
|  | vI (Set) + (1 \| Beaker) | 11 | 1657.60 | 2.69 | 0.19 |
|  | vI (Hg) | 10 | 1659.50 | 4.64 | 0.07 |
|  | vI (Hg) + (1 \| Beaker) | 11 | 1662.20 | 7.33 | 0.02 |
|  | vI (Biofilm) | 10 | 1665.20 | 10.30 | 0.00 |
|  |  |  |  |  |  |
| **Calcium hardness**  **(mg/L)** |  | **K** | **AICc** | **∆AICc** | **wAICc** |
| Fixed effects | **Hardness ~ Set** | 4 | 1816.40 | 0.00 | 0.30 |
|  | Hardness ~ Set + Hg | 5 | 1817.30 | 0.93 | 0.19 |
|  | Hardness ~ Biofilm + Set | 5 | 1818.70 | 2.27 | 0.10 |
|  | Hardness ~ Biofilm:Set | 6 | 1819.00 | 2.64 | 0.08 |
|  | Hardness ~ Hg:Set | 6 | 1819.60 | 3.24 | 0.06 |
| Random effects | **vI (Set)** | 10 | 1671.10 | 0.00 | 0.79 |
|  | vI (Set) + (1 \| Beaker) | 11 | 1673.80 | 2.69 | 0.21 |
|  | vI (Biofilm) | 10 | 1685.00 | 13.90 | 0.00 |
|  | vI (Biofilm) + (1 \| Beaker) | 11 | 1686.30 | 15.11 | 0.00 |
|  | vI (Hg) | 10 | 1690.70 | 19.54 | 0.00 |
|  |  |  |  |  |  |
| **pH** |  | **K** | **AICc** | **∆AICc** | **wAICc** |
| Fixed effects | **pH ~ Biofilm + Set** | 5 | -91.40 | 0.00 | 0.32 |
|  | pH ~ Biofilm + Set + Hg | 6 | -90.30 | 1.11 | 0.18 |
|  | pH ~ Biofilm:Set | 6 | -89.40 | 2.01 | 0.12 |
|  | pH ~ Biofilm:Hg + Set | 7 | -89.00 | 2.48 | 0.09 |
|  | pH ~ Hg:Set + Biofilm | 7 | -88.90 | 2.56 | 0.09 |
| Random effects | **vI (Hg)** | 10 | -46.40 | 0.00 | 0.61 |
|  | vI (Hg) + (1 \| Beaker) | 11 | -43.70 | 2.69 | 0.16 |
|  | vI (Set) | 10 | -42.50 | 3.91 | 0.09 |
|  | vI (Biofilm) | 10 | -42.50 | 3.94 | 0.08 |
|  | vI (Set) + (1 \| Beaker) | 11 | -39.80 | 6.60 | 0.02 |
|  |  |  |  |  |  |
| **Conductivity**  **(mS/cm)** |  | **K** | **AICc** | **∆AICc** | **wAICc** |
| Fixed effects | **Conductivity ~ Biofilm + Set** | 5 | -263.90 | 0.00 | 0.25 |
|  | Conductivity ~ Biofilm:Set | 6 | -263.70 | 0.15 | 0.23 |
|  | Conductivity ~ Biofilm + Set + Hg | 6 | -262.70 | 1.14 | 0.14 |
|  | Conductivity ~ Biofilm:Set + Hg | 7 | -262.50 | 1.39 | 0.13 |
|  | Conductivity ~ Hg:Set + Biofilm | 7 | -261.00 | 2.92 | 0.06 |
| Random effects | **vI (Set)** | 10 | -192.10 | 0.00 | 0.82 |
|  | vI (Set) + (1 \| Beaker) | 11 | -189.00 | 3.01 | 0.18 |
|  | vI (Hg) | 10 | -167.50 | 24.54 | 0.00 |
|  | vI (Biofilm) | 10 | -166.70 | 25.38 | 0.00 |
|  | (1 \| Beaker) | 10 | -166.70 | 25.38 | 0.00 |
|  |  |  |  |  |  |
| **Dissolved oxygen**  **(mg/L)** |  | **K** | **AICc** | **∆AICc** | **wAICc** |
| Fixed effects | **Dissolved oxygen ~ Biofilm:Set** | 6 | 35.40 | 0.00 | 0.50 |
|  | Dissolved oxygen ~ Biofilm:Set + Hg | 7 | 38.00 | 2.59 | 0.14 |
|  | Dissolved oxygen ~ Biofilm:Set + Hg:Set | 8 | 39.10 | 3.72 | 0.08 |
|  | Dissolved oxygen ~ Set | 4 | 39.50 | 4.04 | 0.07 |
|  | Dissolved oxygen ~ Biofilm:Set + Biofilm:Hg | 8 | 39.50 | 4.11 | 0.06 |
| Random effects | **vI (Set)** | 10 | 64.70 | 0.00 | 0.67 |
|  | vI (Set) + (1 \| Beaker) | 11 | 67.80 | 3.08 | 0.14 |
|  | vI (Hg) | 10 | 69.30 | 4.55 | 0.07 |
|  | vI (Biofilm) | 10 | 69.80 | 5.11 | 0.05 |
|  | (1 \| Beaker) | 10 | 70.90 | 6.16 | 0.03 |
|  |  |  |  |  |  |
| **Hg in animals (µg/g)** |  | **K** | **AICc** | **∆AICc** | **wAICc** |
| Fixed effects | Hg _animals_ ~ Biofilm:Set + Hg:Set | 8 | 25.10 | 0.00 | 0.35 |
|  | **Hg _animals_ ~ Hg:Set + Biofilm** | 7 | 25.10 | 0.06 | 0.34 |
|  | Hg _animals_ ~ Biofilm:Hg + Hg:Set | 8 | 26.80 | 1.69 | 0.15 |
|  | Hg _animals_ ~ Biofilm:Set + Biofilm:Hg + Hg:Set | 9 | 26.90 | 1.85 | 0.14 |
|  | Hg _animals_ ~ Biofilm:Set:Hg | 10 | 30.5 | 5.42 | 0.02 |
| Random effects | **vI (Hg)** | 10 | 49.90 | 0.00 | 1.00 |
|  | vI (Set) | 10 | 89.50 | 39.58 | 0.00 |
|  | vI (Biofilm) | 10 | 100.00 | 50.08 | 0.00 |
|  |  |  |  |  |  |
| **Se in animals (µg/g)** |  | **K** | **AICc** | **∆AICc** | **wAICc** |
| Fixed effects | **Se _animals_ ~ Biofilm:Set** | 5 | 93.60 | 0.00 | 0.48 |
|  | Se _animals_ ~ Biofilm:Set + Hg | 6 | 96.10 | 2.49 | 0.14 |
|  | Se _animals_ ~ Biofilm:Set + Hg:Set | 7 | 96.60 | 2.98 | 0.11 |
|  | Se _animals_ ~ Biofilm | 3 | 96.80 | 3.22 | 0.10 |
|  | Se _animals_ ~ Biofilm + Set | 4 | 98.40 | 4.84 | 0.04 |
|  |  |  |  |  |  |
| **Se/Hg molar ratio in animals** |  | **K** | **AICc** | **∆AICc** | **wAICc** |
| Fixed effects | **Se/Hg _animals_ ~ Biofilm:Set:Hg** | 10 | 124.10 | 0.00 | 1.00 |
|  | Se/Hg _animals_ ~ Biofilm:Set+ Biofilm:Hg + Hg:Set | 9 | 143.80 | 19.67 | 0.00 |
|  | Se/Hg _animals_ ~ Biofilm:Set+ Biofilm:Hg | 8 | 153.50 | 29.34 | 0.00 |
|  | Se/Hg _animals_ ~ Biofilm:Set+ Hg:Set | 8 | 157.60 | 33.50 | 0.00 |
|  | Se/Hg _animals_ ~ Biofilm:Set+ Hg | 7 | 161.10 | 36.92 | 0.00 |
| Random effects | **vI (Hg)** | 10 | 124.80 | 0.00 | 1.00 |
|  | vI (Set) | 10 | 173.90 | 49.09 | 0.00 |
|  | vI (Biofilm) | 10 | 176.30 | 51.43 | 0.00 |

**Table A.4.** Summary statistics of fitted final models.

| **Response variable** | **Final model** | **Parameter** | **Estimate ± SE** |
| --- | --- | --- | --- |
| **Hg in animals (µg/g)** | Hg _animals_ ~ Hg:Set + Biofilm + vI (Hg) | Intercept | 4.29 ± 0.28 |
|  |  | Biofilm presence | -0.16 ± 0.04 |
|  |  | Set 2 | 3.84 ± 0.39 |
|  |  | 0.2 µg/L Hg | -3.66 ± 0.28 |
|  |  | 0.2 µg/L Hg:Set 2 | -3.29 ± 0.39 |
|  |  |  |  |
| **Se in animals (µg/g)** | Se _animals_ ~ Biofilm:Set | Intercept | 2.95 ± 0.22 |
|  |  | Biofilm presence | 2.37 ± 0.32 |
|  |  | Set 2 | 0.82 ± 0.32 |
|  |  | Biofilm presence:Set 2 | -1.22 ± 0.45 |
|  |  |  |  |
| **Se/Hg molar ratio in animals** | Se/Hg _animals_ ~ Biofilm:Set:Hg + vI (Hg) | Intercept | 1.78 ± 0.12 |
|  |  | Biofilm presence | 1.71 ± 0.17 |
|  |  | Set 2 | -0.70 ± 0.17 |
|  |  | 0.2 µg/L Hg | 8.92 ± 1.34 |
|  |  | 0.2 µg/L Hg:Biofilm presence | 19.05 ± 1.89 |
|  |  | 0.2 µg/L Hg:Set 2 | -1.22 ± 1.89 |
|  |  | Biofilm presence:Set 2 | -1.23 ± 0.24 |
|  |  | 0.2 µg/L Hg:Biofilm presence:Set 2 | -15.84 ± 2.68 |
|  |  |  |  |
| **Cl in medium (mg/L)** | Cl ~ Set + vI (Set) | Intercept | 633.31 ± 2.05 |
|  |  | Set 2 | -25.88 ± 4.16 |
|  |  |  |  |
| **Calcium hardness (mg/L)** | Hardness ~ Set + vI (Set) | Intercept | 344.92 ± 2.08 |
|  |  | Set 2 | -14.16 ± 5.14 |
|  |  |  |  |
| **Hg in medium (µg/L)** | Hg _medium_ ~ Biofilm:Set:Hg +  vI (Hg) | Intercept | 0.51 ± 0.06 |
|  |  | Biofilm presence | -0.40 ± 0.09 |
|  |  | Set 2 | -0.37 ± 0.09 |
|  |  | 0.2 µg/L Hg | -0.46 ± 0.06 |
|  |  | Biofilm presence:Set 2 | 0.41 ± 0.12 |
|  |  | 0.2 µg/L Hg:Set 2 | 0.35 ± 0.09 |
|  |  | 0.2 µg/L Hg:Biofilm presence | 0.37 ± 0.09 |
|  |  | 0.2 µg/L Hg:Biofilm presence:Set 2 | -0.39 ± 0.12 |
|  |  |  |  |
| **Se in medium (µg/L)** | Se _medium_ ~ Biofilm + vI (Set) | Intercept | 5.89 ± 0.03 |
|  |  | Biofilm presence | -0.14 ± 0.04 |
|  |  |  |  |
| **pH** | pH ~ Biofilm + Set + vI (Hg) | Intercept | 7.8 ± 0.02 |
|  |  | Biofilm presence | -0.1 ± 0.03 |
|  |  | Set 2 | 0.14 ± 0.03 |
|  |  |  |  |
| **Conductivity**  **(mS/cm)** | Conductivity ~ Biofilm + Set +  vI (Set) | Intercept | 2.26 ± 0.003 |
|  |  | Biofilm presence | -0.02 ± 0.004 |
|  |  | Set 2 | -0.1 ± 0.007 |
|  |  |  |  |
| **Dissolved oxygen (mg/L)** | Dissolved oxygen ~ Biofilm:Set + vI (Set) | Intercept | 7.56 ± 0.07 |
|  |  | Biofilm presence | 0.28 ± 0.1 |
|  |  | Set 2 | 0.74 ± 0.11 |
|  |  | Biofilm presence:Set 2 | -0.4 ± 0.15 |
|  |  |  |  |
