# Supplementary material for: Biofilms grown in aquatic microcosms affect mercury and selenium accumulation in Daphnia
Source: Ecotoxicology. 2020 Apr 15;29(4):485–92. doi: 10.1007/s10646-020-02194-4 (PMC7182615; doi:10.1007/s10646-020-02194-4)
Supplement: Supplementary file 2 — Supplementary Appendix 2 [file 10646_2020_2194_MOESM2_ESM.docx]

**Manuscript revision**

**Additional supporting information**

**Biofilms grown in aquatic microcosms affect mercury and selenium accumulation in *Daphnia***

Semona Issa^1*^, Tomasz Maciej Ciesielski^2^, Øyvind Mikkelsen^3^, Sigurd Einum^1^, Veerle L. B. Jaspers^2^

^1^Centre for Biodiversity Dynamics (CBD), Department of Biology, Norwegian University of Science and Technology, Høgskoleringen 5, 7491 Trondheim, Norway

^2^Department of Biology, Norwegian University of Science and Technology, Høgskoleringen 5, 7491 Trondheim, Norway

^3^Department of Chemistry, Norwegian University of Science and Technology, Høgskoleringen 5, 7491 Trondheim, Norway

*E-mail contact: [semona.issa@ntnu.no](mailto:semona.issa@ntnu.no)

**Journal name:** *Ecotoxicology*


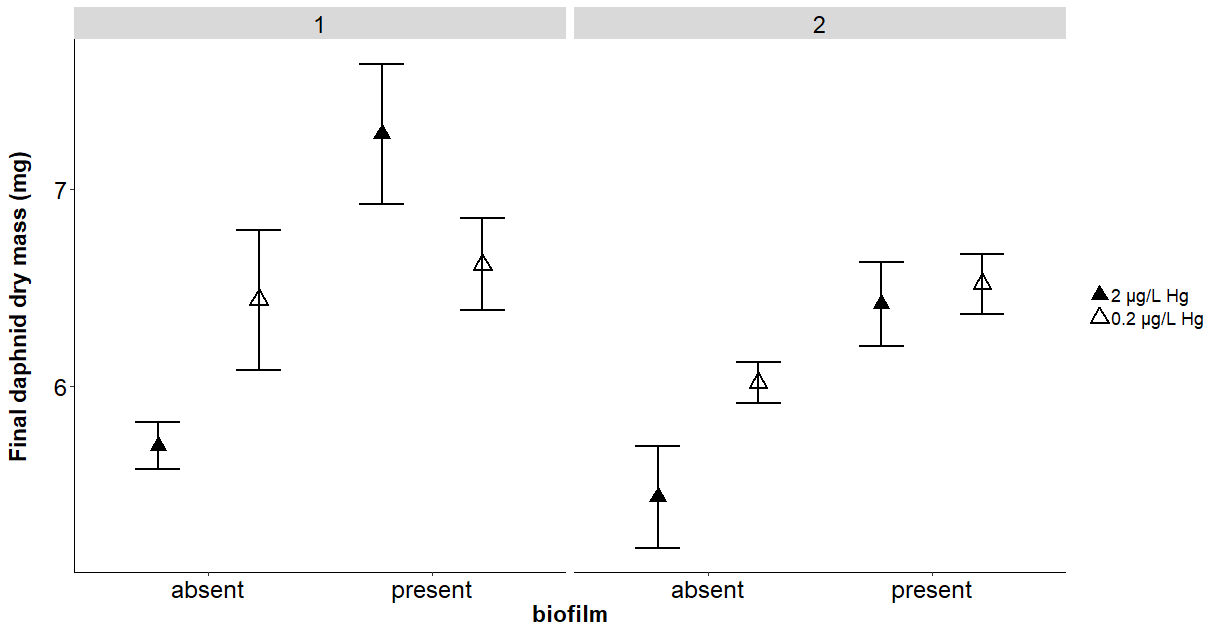


**Fig A1.** Final dry mass of *Daphnia* (mg) in response to growth medium Hg concentrations, biofilm presence versus absence and set (mean ± SE). The y-axis is on a linear scale.

**Table A.3.** Model selection using AICc of candidate models for testing effects of Hg concentration (0.2 μg/L Hg(II) versus 2 μg/L Hg(II)), Biofilm (absent versus present) and Set (1 versus 2) on Hg and Se concentrations in the medium and their content in the animals; Se/Hg molar ratios in the animals; Cl, calcium hardness, pH, conductivity and dissolved oxygen in the medium; and final dry mass of *Daphnia* (mg). Models were sorted by ΔAICc. The best random effect structure was first determined with REML on models that included all listed fixed effects. Fixed effects were then compared with ML using the best random effect structure. K is the number of parameters estimated. The least complex model within 2 ΔAICc is bolded. vI refers to the varIdent function.

| **Response variable** | **Model** | **K** | **AICc** | **∆AICc** | **wAICc** |
| --- | --- | --- | --- | --- | --- |
|  |  |  |  |  |  |
| **Hg in medium (µg/L)** |  |  |  |  |  |
| Fixed effects | **Hg _medium_ ~ Biofilm:Set:Hg** | 10 | -200.20 | 0.00 | 0.94 |
|  | Hg _medium_ ~ Biofilm:Set + Biofilm:Hg + Hg:Set | 9 | -193.20 | 6.98 | 0.03 |
|  | Hg _medium_ ~ Biofilm:Hg + Hg:Set | 8 | -190.50 | 9.65 | 0.01 |
|  | Hg _medium_ ~ Biofilm:Set + Biofilm:Hg | 8 | -190.40 | 9.72 | 0.01 |
|  | Hg _medium_ ~ Biofilm:Set + Hg:Set | 8 | -189.20 | 11.01 | 0.00 |
| Random effects | **vI (Hg)** | 10 | -151.80 | 0.00 | 0.79 |
|  | vI (Hg) + (1 \| Beaker) | 11 | -149.10 | 2.69 | 0.21 |
|  | vI (Biofilm) | 10 | -109.70 | 42.14 | 0.00 |
|  | vI (Biofilm) + (1 \| Beaker) | 11 | -107.00 | 44.83 | 0.00 |
|  | vI (Set) | 10 | -74.00 | 77.78 | 0.00 |
|  |  |  |  |  |  |
| **Se in medium (µg/L)** |  | **K** | **AICc** | **∆AICc** | **wAICc** |
| Fixed effects | **Se _medium_ ~ Biofilm** | 4 | -12.10 | 0.00 | 0.33 |
|  | Se _medium_ ~ Biofilm + Set | 5 | -11.00 | 1.12 | 0.19 |
|  | Se _medium_ ~ Biofilm + Hg | 5 | -10.40 | 1.76 | 0.14 |
|  | Se _medium_ ~ Biofilm + Set + Hg | 6 | -9.20 | 2.93 | 0.08 |
|  | Se ~ Biofilm:Set | 6 | -8.80 | 3.30 | 0.06 |
| Random effects | **vI (Set)** | 10 | 25.20 | 0.00 | 0.69 |
|  | vI (Set) + (1 \| Beaker) | 11 | 27.90 | 2.69 | 0.18 |
|  | vI (Hg) | 10 | 29.70 | 4.52 | 0.07 |
|  | vI (Biofilm) | 10 | 32.00 | 6.87 | 0.02 |
|  | vI (Hg) + ( 1\| Beaker) | 11 | 32.40 | 7.22 | 0.02 |
|  |  |  |  |  |  |
| **Cl in medium (mg/L)** |  | **K** | **AICc** | **∆AICc** | **wAICc** |
| Fixed effects | **Cl ~ Set** | 4 | 1794.20 | 0.00 | 0.44 |
|  | Cl ~ Biofilm + Set | 5 | 1796.30 | 2.04 | 0.16 |
|  | Cl ~ Set + Hg | 5 | 1796.40 | 2.20 | 0.14 |
|  | Cl ~ Biofilm:Set | 6 | 1797.60 | 3.43 | 0.08 |
|  | Cl ~ Biofilm + Set + Hg | 6 | 1798.50 | 4.30 | 0.05 |
| Random effects | **vI (Set)** | 10 | 1654.90 | 0.00 | 0.72 |
|  | vI (Set) + (1 \| Beaker) | 11 | 1657.60 | 2.69 | 0.19 |
|  | vI (Hg) | 10 | 1659.50 | 4.64 | 0.07 |
|  | vI (Hg) + (1 \| Beaker) | 11 | 1662.20 | 7.33 | 0.02 |
|  | vI (Biofilm) | 10 | 1665.20 | 10.30 | 0.00 |
|  |  |  |  |  |  |
| **Calcium hardness**  **(mg/L)** |  | **K** | **AICc** | **∆AICc** | **wAICc** |
| Fixed effects | **Hardness ~ Set** | 4 | 1816.40 | 0.00 | 0.30 |
|  | Hardness ~ Set + Hg | 5 | 1817.30 | 0.93 | 0.19 |
|  | Hardness ~ Biofilm + Set | 5 | 1818.70 | 2.27 | 0.10 |
|  | Hardness ~ Biofilm:Set | 6 | 1819.00 | 2.64 | 0.08 |
|  | Hardness ~ Hg:Set | 6 | 1819.60 | 3.24 | 0.06 |
| Random effects | **vI (Set)** | 10 | 1671.10 | 0.00 | 0.79 |
|  | vI (Set) + (1 \| Beaker) | 11 | 1673.80 | 2.69 | 0.21 |
|  | vI (Biofilm) | 10 | 1685.00 | 13.90 | 0.00 |
|  | vI (Biofilm) + (1 \| Beaker) | 11 | 1686.30 | 15.11 | 0.00 |
|  | vI (Hg) | 10 | 1690.70 | 19.54 | 0.00 |
|  |  |  |  |  |  |
| **pH** |  | **K** | **AICc** | **∆AICc** | **wAICc** |
| Fixed effects | **pH ~ Biofilm + Set** | 5 | -91.40 | 0.00 | 0.32 |
|  | pH ~ Biofilm + Set + Hg | 6 | -90.30 | 1.11 | 0.18 |
|  | pH ~ Biofilm:Set | 6 | -89.40 | 2.01 | 0.12 |
|  | pH ~ Biofilm:Hg + Set | 7 | -89.00 | 2.48 | 0.09 |
|  | pH ~ Hg:Set + Biofilm | 7 | -88.90 | 2.56 | 0.09 |
| Random effects | **vI (Hg)** | 10 | -46.40 | 0.00 | 0.61 |
|  | vI (Hg) + (1 \| Beaker) | 11 | -43.70 | 2.69 | 0.16 |
|  | vI (Set) | 10 | -42.50 | 3.91 | 0.09 |
|  | vI (Biofilm) | 10 | -42.50 | 3.94 | 0.08 |
|  | vI (Set) + (1 \| Beaker) | 11 | -39.80 | 6.60 | 0.02 |
|  |  |  |  |  |  |
| **Conductivity**  **(mS/cm)** |  | **K** | **AICc** | **∆AICc** | **wAICc** |
| Fixed effects | **Conductivity ~ Biofilm + Set** | 5 | -263.90 | 0.00 | 0.25 |
|  | Conductivity ~ Biofilm:Set | 6 | -263.70 | 0.15 | 0.23 |
|  | Conductivity ~ Biofilm + Set + Hg | 6 | -262.70 | 1.14 | 0.14 |
|  | Conductivity ~ Biofilm:Set + Hg | 7 | -262.50 | 1.39 | 0.13 |
|  | Conductivity ~ Hg:Set + Biofilm | 7 | -261.00 | 2.92 | 0.06 |
| Random effects | **vI (Set)** | 10 | -192.10 | 0.00 | 0.82 |
|  | vI (Set) + (1 \| Beaker) | 11 | -189.00 | 3.01 | 0.18 |
|  | vI (Hg) | 10 | -167.50 | 24.54 | 0.00 |
|  | vI (Biofilm) | 10 | -166.70 | 25.38 | 0.00 |
|  | (1 \| Beaker) | 10 | -166.70 | 25.38 | 0.00 |
|  |  |  |  |  |  |
| **Dissolved oxygen**  **(mg/L)** |  | **K** | **AICc** | **∆AICc** | **wAICc** |
| Fixed effects | **Dissolved oxygen ~ Biofilm:Set** | 6 | 35.40 | 0.00 | 0.50 |
|  | Dissolved oxygen ~ Biofilm:Set + Hg | 7 | 38.00 | 2.59 | 0.14 |
|  | Dissolved oxygen ~ Biofilm:Set + Hg:Set | 8 | 39.10 | 3.72 | 0.08 |
|  | Dissolved oxygen ~ Set | 4 | 39.50 | 4.04 | 0.07 |
|  | Dissolved oxygen ~ Biofilm:Set + Biofilm:Hg | 8 | 39.50 | 4.11 | 0.06 |
| Random effects | **vI (Set)** | 10 | 64.70 | 0.00 | 0.67 |
|  | vI (Set) + (1 \| Beaker) | 11 | 67.80 | 3.08 | 0.14 |
|  | vI (Hg) | 10 | 69.30 | 4.55 | 0.07 |
|  | vI (Biofilm) | 10 | 69.80 | 5.11 | 0.05 |
|  | (1 \| Beaker) | 10 | 70.90 | 6.16 | 0.03 |
|  |  |  |  |  |  |
| **Hg in animals (µg/g)** |  | **K** | **AICc** | **∆AICc** | **wAICc** |
| Fixed effects | Hg _animals_ ~ Biofilm:Set + Hg:Set | 8 | 25.10 | 0.00 | 0.35 |
|  | **Hg _animals_ ~ Hg:Set + Biofilm** | 7 | 25.10 | 0.06 | 0.34 |
|  | Hg _animals_ ~ Biofilm:Hg + Hg:Set | 8 | 26.80 | 1.69 | 0.15 |
|  | Hg _animals_ ~ Biofilm:Set + Biofilm:Hg + Hg:Set | 9 | 26.90 | 1.85 | 0.14 |
|  | Hg _animals_ ~ Biofilm:Set:Hg | 10 | 30.5 | 5.42 | 0.02 |
| Random effects | **vI (Hg)** | 10 | 49.90 | 0.00 | 1.00 |
|  | vI (Set) | 10 | 89.50 | 39.58 | 0.00 |
|  | vI (Biofilm) | 10 | 100.00 | 50.08 | 0.00 |
|  |  |  |  |  |  |
| **Se in animals (µg/g)** |  | **K** | **AICc** | **∆AICc** | **wAICc** |
| Fixed effects | **Se _animals_ ~ Biofilm:Set** | 5 | 93.60 | 0.00 | 0.48 |
|  | Se _animals_ ~ Biofilm:Set + Hg | 6 | 96.10 | 2.49 | 0.14 |
|  | Se _animals_ ~ Biofilm:Set + Hg:Set | 7 | 96.60 | 2.98 | 0.11 |
|  | Se _animals_ ~ Biofilm | 3 | 96.80 | 3.22 | 0.10 |
|  | Se _animals_ ~ Biofilm + Set | 4 | 98.40 | 4.84 | 0.04 |
|  |  |  |  |  |  |
| **Se/Hg molar ratio in animals** |  | **K** | **AICc** | **∆AICc** | **wAICc** |
| Fixed effects | **Se/Hg _animals_ ~ Biofilm:Set:Hg** | 10 | 124.10 | 0.00 | 1.00 |
|  | Se/Hg _animals_ ~ Biofilm:Set+ Biofilm:Hg + Hg:Set | 9 | 143.80 | 19.67 | 0.00 |
|  | Se/Hg _animals_ ~ Biofilm:Set+ Biofilm:Hg | 8 | 153.50 | 29.34 | 0.00 |
|  | Se/Hg _animals_ ~ Biofilm:Set+ Hg:Set | 8 | 157.60 | 33.50 | 0.00 |
|  | Se/Hg _animals_ ~ Biofilm:Set+ Hg | 7 | 161.10 | 36.92 | 0.00 |
| Random effects | **vI (Hg)** | 10 | 124.80 | 0.00 | 1.00 |
|  | vI (Set) | 10 | 173.90 | 49.09 | 0.00 |
|  | vI (Biofilm) | 10 | 176.30 | 51.43 | 0.00 |
|  |  |  |  |  |  |
| **Final *Daphnia***  **dry mass (mg)** |  |  |  |  |  |
| Fixed effects | **Dry mass ~ Biofilm:Hg + Set** | 6 | 73.3 | 0.00 | 0.43 |
|  | Dry mass ~ Biofilm:Hg + Hg:Set | 7 | 75.3 | 2.06 | 0.15 |
|  | Dry mass ~ Biofilm:Set + Biofilm:Hg | 7 | 76.0 | 2.76 | 0.11 |
|  | Dry mass ~ Biofilm:Hg | 5 | 76.6 | 3.34 | 0.08 |
|  | Dry mass ~ Biofilm + Set | 4 | 76.9 | 3.61 | 0.07 |

**Table A.4.** Summary statistics of fitted final models.

| **Response variable** | **Final model** | **Parameter** | **Estimate ± SE** |
| --- | --- | --- | --- |
| **Hg in animals (µg/g)** | Hg _animals_ ~ Hg:Set + Biofilm + vI (Hg) | Intercept | 4.29 ± 0.28 |
|  |  | Biofilm presence | -0.16 ± 0.04 |
|  |  | Set 2 | 3.84 ± 0.39 |
|  |  | 0.2 µg/L Hg | -3.66 ± 0.28 |
|  |  | 0.2 µg/L Hg:Set 2 | -3.29 ± 0.39 |
|  |  |  |  |
| **Se in animals (µg/g)** | Se _animals_ ~ Biofilm:Set | Intercept | 2.95 ± 0.22 |
|  |  | Biofilm presence | 2.37 ± 0.32 |
|  |  | Set 2 | 0.82 ± 0.32 |
|  |  | Biofilm presence:Set 2 | -1.22 ± 0.45 |
|  |  |  |  |
| **Se/Hg molar ratio in animals** | Se/Hg _animals_ ~ Biofilm:Set:Hg + vI (Hg) | Intercept | 1.78 ± 0.12 |
|  |  | Biofilm presence | 1.71 ± 0.17 |
|  |  | Set 2 | -0.70 ± 0.17 |
|  |  | 0.2 µg/L Hg | 8.92 ± 1.34 |
|  |  | 0.2 µg/L Hg:Biofilm presence | 19.05 ± 1.89 |
|  |  | 0.2 µg/L Hg:Set 2 | -1.22 ± 1.89 |
|  |  | Biofilm presence:Set 2 | -1.23 ± 0.24 |
|  |  | 0.2 µg/L Hg:Biofilm presence:Set 2 | -15.84 ± 2.68 |
|  |  |  |  |
| **Cl in medium (mg/L)** | Cl ~ Set + vI (Set) | Intercept | 633.31 ± 2.05 |
|  |  | Set 2 | -25.88 ± 4.16 |
|  |  |  |  |
| **Calcium hardness (mg/L)** | Hardness ~ Set + vI (Set) | Intercept | 344.92 ± 2.08 |
|  |  | Set 2 | -14.16 ± 5.14 |
|  |  |  |  |
| **Hg in medium (µg/L)** | Hg _medium_ ~ Biofilm:Set:Hg +  vI (Hg) | Intercept | 0.51 ± 0.06 |
|  |  | Biofilm presence | -0.40 ± 0.09 |
|  |  | Set 2 | -0.37 ± 0.09 |
|  |  | 0.2 µg/L Hg | -0.46 ± 0.06 |
|  |  | Biofilm presence:Set 2 | 0.41 ± 0.12 |
|  |  | 0.2 µg/L Hg:Set 2 | 0.35 ± 0.09 |
|  |  | 0.2 µg/L Hg:Biofilm presence | 0.37 ± 0.09 |
|  |  | 0.2 µg/L Hg:Biofilm presence:Set 2 | -0.39 ± 0.12 |
|  |  |  |  |
| **Se in medium (µg/L)** | Se _medium_ ~ Biofilm + vI (Set) | Intercept | 5.89 ± 0.03 |
|  |  | Biofilm presence | -0.14 ± 0.04 |
|  |  |  |  |
| **pH** | pH ~ Biofilm + Set + vI (Hg) | Intercept | 7.8 ± 0.02 |
|  |  | Biofilm presence | -0.1 ± 0.03 |
|  |  | Set 2 | 0.14 ± 0.03 |
|  |  |  |  |
| **Conductivity**  **(mS/cm)** | Conductivity ~ Biofilm + Set +  vI (Set) | Intercept | 2.26 ± 0.003 |
|  |  | Biofilm presence | -0.02 ± 0.004 |
|  |  | Set 2 | -0.1 ± 0.007 |
|  |  |  |  |
| **Dissolved oxygen (mg/L)** | Dissolved oxygen ~ Biofilm:Set + vI (Set) | Intercept | 7.56 ± 0.07 |
|  |  | Biofilm presence | 0.28 ± 0.1 |
|  |  | Set 2 | 0.74 ± 0.11 |
|  |  | Biofilm presence:Set 2 | -0.4 ± 0.15 |
|  |  |  |  |
| **Final *Daphnia***  **dry mass (mg)** | Dry mass ~ Biofilm:Hg + Set | Intercept | 5.77 ± 0.19 |
|  |  | Set 2 | -0.41 ± 0.17 |
|  |  | Biofilm presence | 1.28 ± 0.24 |
|  |  | 0.2 µg/L Hg | 0.66 ± 0.24 |
|  |  | 0.2 µg/L Hg:Biofilm presence | -0.94 ± 0.34 |

**Table A.5.** Exposure variable averages on the first and last experimental days are compared across all sets and treatment combinations. Values are given as mean ± SE.

|  |  | **Treatment** | | | | | | | |
| --- | --- | --- | --- | --- | --- | --- | --- | --- | --- |
|  |  | Biofilm present  0.2 Hg (μg/L) | | Biofilm absent  0.2 Hg (μg/L) | | Biofilm present  2 Hg (μg/L) | | Biofilm absent  2 Hg (μg/L) | |
|  |  |  | |  | |  | |  | |
|  |  | Day 1 | Day 3 | Day 1 | Day 3 | Day 1 | Day 3 | Day 1 | Day 3 |
|  | **Set** |  |  |  |  |  |  |  |  |
| **Hg^2+^ (μg/L)** | 1 | 0,026  ±  0,003 | 0,021  ±  0,005 | 0,076  ±  0,007 | 0,023  ±  0,01 | 0,15  ±  0,01 | 0,080  ±  0,01 | 0,84  ±  0,02 | 0,19  ±  0,08 |
|  | 2 | 0,027  ±  0,004 | 0,025  ±  0,005 | 0,028  ±  0,009 | 0,026  ±  0,01 | 0,11  ±  0,01 | 0,20  ±  0,006 | 0,20  ±  0,05 | 0,080  ±  0,03 |
|  |  |  |  |  |  |  |  |  |  |
| **Se^2-^ (μg/L)** | 1 | 5,8  ±  0,09 | 5,7  ±  0,04 | 5,8  ±  0,04 | 6,0  ±  0,06 | 5,7  ±  0,1 | 5,7  ±  0,06 | 5,9  ±  0,04 | 6,0  ±  0,08 |
|  | 2 | 5,9  ±  0,05 | 5,5  ±  0,2 | 5,7  ±  0,06 | 6,2  ±  0,1 | 5,8  ±  0,06 | 5,6  ±  0,1 | 5,7  ±  0,07 | 5,7  ±  0,06 |
